# Handgrip strength values of Portuguese older adults: a population based study

**DOI:** 10.1186/s12877-017-0590-5

**Published:** 2017-08-23

**Authors:** Joana Mendes, Teresa F. Amaral, Nuno Borges, Alejandro Santos, Patrícia Padrão, Pedro Moreira, Cláudia Afonso, Rita Negrão

**Affiliations:** 1 Department of Biomedicine, Biochemistry Unit, Faculty of Medicine, University of Porto, Rua Dr. Plácido da Costa, 4200-450 Porto, Portugal; 2 I3S – Institute for Research and Innovation in Health, University of Porto, Rua Alfredo Allen, 208, 4200-135 Porto, Portugal; 3 Faculty of Nutrition and Food Sciences, University of Porto, Rua Dr. Roberto Frias, 4200-465 Porto, Portugal; 4 UISPA-IDMEC, Faculty of Engineering, University of Porto, Rua Dr. Roberto Frias, 4200-465 Porto, Portugal; 5 CINTESIS - Centre for Health Technology and Services Research, Rua Dr. Plácido da Costa, 4200-450 Porto, Portugal; 6 EPIUnit, Institute of Public Health, University of Porto, Rua das Taipas, n° 135, 4050-600 Porto, Portugal; 7 The Research Centre in Physical Activity, Health and Leisure, University of Porto, Rua Dr. Plácido Costa, 91, 4200-450 Porto, Portugal

**Keywords:** Aged, Hand strength, Health status, Muscle strength dynamometer, Population characteristics

## Abstract

**Background:**

Handgrip strength is used to identify sarcopenia and frailty phenotypes, being a potential predictor of mortality in older adults. However, uniformity is lacking in the reference values. This study aimed to describe handgrip strength values of older population aged ≥65 years in Portugal, considering the possible influence of anthropometric parameters.

**Methods:**

A cross-sectional study was conducted in Portugal, among 1500 older adults aged ≥65 years old, according to “The Nutrition UP 65 Study Protocol”. Demographic data were collected and cognitive performance, subjective general health, physical activity, anthropometric parameters and nutritional status were assessed and analysed. Handgrip strength data was obtained with a Jamar dynamometer. A Pubmed/Medline search was carried out to compare handgrip strength data between Portuguese older adults and other older populations.

**Results:**

Handgrip strength was higher among men than among women (30.3 ± 9.2 Kgf vs 18 ± 5.4 Kgf, *p* < 0.001, respectively). In general, handgrip strength values of Portuguese older adults were lower than other older populations. In our sample, age, cognitive and nutritional status, self-reported sitting time and practice of physical activity were significantly correlated with handgrip strength in both sexes. Concerning anthropometric parameters, height was the most significantly correlated with handgrip strength (*r* = 0.34, *p* < 0.001, in women and *r* = 0.40, *p* < 0.001, in men).

**Conclusion:**

This study described, for the first time, handgrip strength values of Portuguese population aged ≥65 years, according to age and to sex-specific tertiles of height. The definition of handgrip strength reference values in this age group merits further reflection.

## Background

Handgrip strength (HGS) is a marker of overall body muscle strength and is used to identify sarcopenia and frailty phenotypes [1].

At hospital admission, lower HGS was associated with a decreased probability of discharge alive over time [2], moreover in community-dwelling older adults, HGS was a potential predictor of mortality (multivariate-adjusted relative risk of all causes of death for the lowest HGS quintile: 1.38, 95% CI 1.01–1.88, in men and 1.54, 95% CI 1.20–1.98, in women) [3]. Muscle strength is an indicator of muscle quality has shown to be more significant than muscle mass in estimating mortality risk [4]. One possible explanation is that loss of motor neurons with age leads to a rise in size of remaining motor units, but with higher preservation of type 1 fibers, resulting in conservation of mass with relatively less type 2 fibers, and thus, lower strength [5].

According to recent demographic projections, the world population is rapidly getting older [6, 7]. The European Union population aged ≥65 years is estimated to rise by over 25% by 2035 [6, 7]. In this context, it has been reported that HGS reference values for older adults are required [8], but HGS data have not yet been described for the Portuguese population. The use of other populations as reference may be unsuitable, because there are variations in the skeletal muscle mass and function from individuals from different ethnicities [9].

The aim of this study was to describe HGS values of older population aged ≥65 years in Portugal, considering the possible influence of anthropometric parameters.

## Methods

### Study and sample design

A cross-sectional study was carried out in Portugal, in a cluster sample of 1500 older adults ≥65 years old, between December 2015 and June 2016, according to “The Nutrition UP 65 Study Protocol” [7].

Data from the 2011 Census was used to gather a nationally representative sample of Portuguese older adults in terms of sex, age, educational level and residence area [7, 10]. The regional areas were defined based on Nomenclature of Territorial Units for Statistics [7]. A random, stratified and cluster sampling approach was used. In each regional area, ≥ 3 town councils with >250 inhabitants were randomly identified. The potential participants were contacted through by phone, home approach or via institutions, such as parish centres and town councils. Individuals were considered eligible if they only had Portuguese nationality with current tax residence in Portugal and if they were aged ≥65 years. Exclusion criteria were having upper limb deformities or being unable to perform HGS measurements, due to conditions that lead to incapacity to understand the explanations and to carry out the technique correctly.

The initial sample was composed of 5% of older adults in nursing homes, the proportion that was described for the Portuguese population [10]. Participants were considered community-dwelling individuals if they slept in their own residence or in the house of a relative or a friend more than half the preceding month.

### Data collection and variable definition

Eight interviewers collected data during the study period. In order to improve inter and intra rater agreement, all interviewers were previously trained and the corresponding errors were obtained for anthropometric values. Intra-rater error ranged from 0.05 to 0.34% and inter-rater error varied between 0.19 and 1.48%. For trained anthropometrists, these values are considered acceptable [7].

Demographic data included information on sex, date of birth, marital status, education and residence area. The following age categories were considered: [65–75[, [75–85[and ≥85 years old. Educational level was ascertained by the years of completed schooling. Three education categories were constituted: no formal schooling, 1 to 4 years of completed schooling and >4 years of completed schooling.

Cognitive performance was assessed and classified according to the Mini Mental State Examination (MMSE) - Portuguese version [7, 11]. According to this version of the MMSE, the cut-off scores for “cognitive impairment” are: individuals without education, < 15 points; 1 to 11 years of schooling, < 22 points; and >11 years of schooling, < 27 points [7, 11].

Data on subjective general health, related to chronic diseases, were gathered using questions from 2005 to 2006 Portuguese National Health Survey [7, 12]. Questions included self-reported diagnosis of chronic diseases in the past 12 months, as follows: the presence of depression, chronic neck problems or neck pain, chronic lumbar problems or lumbar pain, arthrosis, asthma and allergies, chronic bronchitis or emphysema or chronic obstructive pulmonary disease, myocardial infarction or its chronic consequences, angina pectoris or coronary heart disease, hypertension, stroke or its chronic consequences, diabetes, hepatic cirrhosis, bladder control problems or urinary incontinence and chronic renal disease [12].

Physical activity during the previous 7 days was assessed by the Short Form - International Physical Activity Questionnaire, namely on how much time the participant spent sitting and how much time the individual spent walking or practicing some type of activity, not sitting or lying down, such as gardening, agriculture, gym or other activity [7, 13].

Anthropometric measurements and nutritional status were evaluated as described in “The Nutrition UP 65 Study Protocol” [7]. Anthropometric measurements included weight, height, triceps skinfold thickness, mid-upper arm, waist and calf circumferences. According to the study sample, the following sex-specific tertiles of height were considered: < 148; [148–153[and ≥153 cm, in women and <161; [161–167[and ≥167 cm, in men. Body mass index (BMI) was calculated (BMI = weight [kg]/(height [m])^2^). Mid-arm muscle circumference was estimated from mid-arm circumference and triceps skinfold thickness [7]. The Mini-Nutritional Assessment - Short Form (MNA-SF) was applied to identify participants at risk of undernutrition and undernourished. Participants were considered undernourished if the final score was ≤7 points and they were considered at risk of undernutrition if the final score was between 8 and 11 points. Participants with a score ≥ 12 points were classified without undernutrition risk/undernutrition [7, 14].

### Handgrip strength

Handgrip strength data was obtained with a Jamar Plus® + Digital Hand Dynamometer (Sammons Preston Inc., Bolingbrook, Illinois, USA) calibrated by the manufacturer, with a resolution of 0.1 Kgf. Measurements were carried out with the subject seated, shoulders neutrally rotated and adducted, elbow flexed at 90°, forearm in neutral and wrist between 0 and 30° of dorsiflexion, as recommended by the American Society of Hand Therapists and described in “The Nutrition UP 65 Study Protocol” [7, 15]. Each participant carried out three measurements with a pause of 1 min between them [7, 15, 16]. The maximum value of three consecutive measurements with the non-dominant hand was registered [7, 17–19]. The dominant hand was used (1.27% of cases) when the individual was unable to conduct the measurement with the non-dominant hand. Handgrip strength was measured in 1496 (99.7%) participants of the total sample, because there were four (0.27%) missing records.

### Comparison with data previously published

To compare present data with HGS previously published, a Pubmed/Medline search was performed and the key words “hand strength” and “reference values” were used. Data were selected for comparison if the mean values of HGS were presented stratified by sex and age, as well as if the type of dynamometer and the technique of measurement were described. Studies in which participants were all under 65 years old and studies performed in clinical settings were excluded. A total of 15 studies were analyzed.

### Statistical analysis

Characteristics of the sample were presented stratified by sex and age. Categorical variables were summarized as counts and proportions and compared using the chi-square test. The normality of variables distribution was verified with the Kolmogorov-Smirnov test. Means and their standard deviation values were presented and were compared with ANOVA test. For MMSE, median and interquartile range were also calculated.

For the studied variables Pearson’s correlation coefficients were calculated. Descriptive values of HGS, including 10, 15, 25, 50, 75, 85 and 90 percentiles, and 85% value of HGS mean were calculated and stratified by age and by sex-specific tertiles of height.

Handgrip strength mean differences between other older populations and our sample were quantified, according to the available age ranges. Values of non-dominant hand were considered whenever possible. In studies whose HGS values were presented for each hand separately (“left” and “right”), the average of values for both hands was used for comparison with our sample. Portuguese data were graphically compared with Danish and United Kingdom data, stratified according to height ranges.

All the statistical analysis was carried out using Statistical Package for Social Sciences for Windows (SPSS, version 24.0). Results were considered significant when *p* < 0.05.

## Results

Characteristics of the sample are summarized in Table 1. Age ranged from 65 to 100 years, with a mean of 75 ± 7.1 years. Women represented a slightly higher proportion of the sample (58%). Most of the participants were from the Centre of Portugal (51.6%) and approximately half of them were married (44.5%).

**Table 1:** Characteristics of the sample according to sex and to age range

	Women				Men			
	Age range (years)			*p*	Age range (years)			*p*
	[65–75[	[75–85[	≥85		[65–75[	[75–85[	≥85	
*n* (%)	423 (48.7)	328 (37.8)	117 (13.5)		354 (56.4)	218 (34.7)	56 (8.9)	
Education (years), *n* (%)								
no formal schooling	28 (6.6)	84 (25.6)	40 (34.2)		17 (4.8)	32 (14.7)	11 (19.6)	
1–4 schooling years	355 (83.9)	228 (69.5)	73 (62.4)	<0.001	295 (83.3)	169 (77.5)	39 (69.7)	<0.001
˃ 4 schooling years	40 (9.5)	16 (4.9)	4 (3.4)		42 (11.9)	17 (7.8)	6 (10.7)	
Mini Mental State Examination, *n* (%)								
no cognitive impairment	410 (96.9)	296 (90.2)	98 (83.8)	<0.001	316 (89.3)	189 (86.7)	45 (80.4)	0.152
cognitive impairment^a^	13 (3.1)	32 (9.8)	19 (16.2)		38 (10.7)	29 (13.3)	11 (19.6)	
Number of chronic diseases^b^, mean (SD)	4.1 (2.2)	4.5 (2.1)	4.2 (2.0)	0.129	3.1 (1.9)	3.1 (1.7)	3.0 (2.1)	0.947
Self-reported sitting time (hours/day), mean (SD)	4.6 (2.7)	6.3 (3.1)	7.6 (3.5)	<0.001	4.9 (2.8)	5.5 (2.9)	6.9 (3.4)	<0.001
Practice of physical activity (hours/day), mean (SD)	1.9 (2.1)	0.9 (1.1)	0.6 (0.9)	0.523	1.9 (2.0)	1.4 (1.9)	0.9 (1.1)	0.264
Weight (Kg), mean (SD)	70.3 (12.8)	68.6 (12.6)	62.3 (11.2)	0.421	78.1 (12.2)	77.5 (11.8)	72.6 (9.8)	0.810
Height (cm), mean (SD)	152.9 (5.9)	150.5 (5.9)	147.3 (5.7)	0.006	165.9 (6.8)	163.9 (6.6)	161.5 (6.6)	0.232
Body mass index (Kg/m^2^), mean (SD)	30.0 (5.0)	30.2 (5.1)	28.6 (4.6)	0.044	28.3 (3.9)	28.8 (4.2)	27.9 (3.9)	0.197
Mid-arm muscle circumference^c^ (cm), mean (SD)	22.8 (3.8)	22.6 (2.9)	22.2 (2.9)	0.683	24.8 (3.1)	24.6 (6.5)	22.9 (3.0)	0.013
Waist circumference^d^ (cm), mean (SD)	96.4 (12.0)	98.9 (12.4)	95.9 (11.8)	0.212	101.9 (10.6)	104.2 (10.2)	102.8 (10.9)	0.895
Calf circumference (cm), mean (SD)	35.9 (3.9)	35.5 (3.5)	33.8 (3.1)	<0.001	36.2 (3.6)	35.9 (3.2)	34.6 (2.7)	0.007
Mini Nutritional Assessment – Short Form, *n* (%)								
without undernutrition	357 (84.4)	266 (81.1)	83 (70.9)	0.004	316 (89.3)	189 (86.7)	45 (80.4)	0.152
with risk of undernutrition or undernourished	66 (15.6)	62 (18.9)	34 (29.1)		38 (10.7)	29 (13.3)	11 (19.6)	
Handgrip strength (Kgf), mean (SD)	20.1 (5.4)	16.6 (4.6)	14.3 (3.9)	<0.001	33.4 (9.3)	27.4 (7.3)	22.5 (7.2)	0.006

^a^The cut-off scores for “cognitive impairment” are as follows: individuals with no education, <15 points; 1 to 11 years of school completed, <22 points; and >11 years of school completed, <27 points

^b^Information was not reported by one individual (0.06%)

^c^Information was not obtained for six individuals (0.4%)

^d^Information was not collected for 12 individuals (0.8%). *Abbreviations*: SD, standard deviation

Mini Mental State Examination ranged from 10 to 30 points in total sample. According to cut-offs of the Portuguese version of MMSE, scores are presented by mean ± standard deviation; median (25th percentile-75th percentile) as follow: [12 ± 1.7; 13 (11–14) in “cognitive impairment” and 22 ± 0.3; 22 (19–25) in “no cognitive impairment”, if participants had no education]; [18 ± 3.2; 19 (16–21) in “cognitive impairment” and 28 ± 2; 28 (26–29) in “no cognitive impairment”, if participants had 1 to 11 years of school completed]; [26 ± 1.2; 26 (25–26) in “cognitive impairment” and 29 ± 0.8; 29 (29–30) in “no cognitive impairment”, if participants had >11 years of school completed].

In total sample, chronic diseases were presented in the following proportions of cases: depression (19.9%), chronic neck problems or neck pain (50.1%), chronic lumbar problems or lumbar pain (61.9%), arthrosis (59.7%), asthma and allergies (8.6%), chronic bronchitis or emphysema or chronic obstructive pulmonary disease (15.8%), myocardial infarction or its chronic consequences (3.7%), angina pectoris or coronary heart disease (17.1%), hypertension (63.9%), stroke or its chronic consequences (7.1%), diabetes (27.5%), hepatic cirrhosis (3.9%), bladder control problems or urinary incontinence (12.1%), chronic renal disease (9.1%).

Height ranged from 131.8 to 170 cm in women, with a mean of 151.2 ± 6.2 cm, and from 140.3 to 185 cm in men, with a mean of 164.8 ± 6.8 cm. Participants showed an average of BMI of 29.9 ± 5 kg/m^2^ in women and of 28.5 ± 4 kg/m^2^ in men. According to the MNA-SF, 1.3% of women and 1.3% of men were classified as undernourished, and 17.4% of women, as well as 11.1% of men were classified at nutritional risk.

Handgrip strength was higher among men than among women (30.3 ± 9.2 Kgf vs 18 ± 5.4 Kgf, *p* < 0.001). Handgrip strength values of Portuguese older adults, stratified by age and by sex-specific tertiles of height were described in Table 2.

**Table 2:** Values of handgrip strength of Portuguese older women and men, stratified by age and height

Age range (years)	Height range (cm)	n (%)	Handgrip strength (Kgf)									
			mean (SD)	85% of mean	min-max	P10	P15	P25	P50	P75	P85	P90
Women, *n* = 868												
[65–75[	<148	97 (11.2)	18.7 (4.6)	15.9	7.9–30.1	12.6	14.1	16.3	18.1	21.9	22.9	25.1
	[148–153[	154 (17.7)	19.8 (5.5)	16.8	3.8–32.9	12.5	14.3	16.9	20.5	23.1	24.6	25.9
	≥153	172 (19.8)	21.1 (5.5)	17.9	9.6–35.5	14.3	15.2	17.0	21.0	25.4	27.0	28.3
[75–85[	<148	122 (14.1)	15.3 (4.1)	13.0	4.8–25.8	10.2	10.9	12.7	15.1	17.9	19.8	20.7
	[148–153[	109 (12.5)	16.8 (4.7)	14.3	4.3–28.2	9.9	12.1	14.3	16.5	19.9	22.1	22.9
	≥153	97 (11.2)	17.9 (4.7)	15.2	6.3–30.7	11.8	12.8	15.5	17.6	21.6	23.0	23.7
≥85	<148	70 (8.1)	13.4 (3.8)	11.4	6.0–24.3	8.6	9.4	10.5	13.3	15.9	17.5	18.3
	[148–153[	28 (3.2)	14.8 (3.7)	12.6	6.7–21.1	9.6	10.2	11.1	15.1	17.7	19.1	19.5
	≥153	19 (2.2)	16.9 (3.9)	14.4	9.1–22.8	11.3	12.2	14.4	18.0	19.4	22.1	22.7
Men, *n* = 628												
[65–75[	<161	92 (14.6)	28.6 (7.9)	24.3	9.6–48.0	16.9	18.8	23.7	29.3	34.5	35.1	38.2
	[161–167[	118 (18.8)	32.6 (8.4)	27.7	11.2–51.4	20.5	23.8	26.3	32.8	38.9	41.8	43.8
	≥167	144 (22.9)	36.9 (9.2)	31.4	9.4–58.9	23.9	27.3	31.1	38.5	43.9	45.8	47.3
[75–85[	<161	86 (13.7)	25.5 (7.7)	21.7	2.3–41.5	16.3	17.4	20.8	25.9	30.1	33.6	34.9
	[161–167[	77 (12.3)	27.5 (6.8)	23.4	5.2–46.4	19.4	20.4	23.6	27.4	32.1	34.0	35.2
	≥167	55 (8.8)	30.4 (6.4)	25.8	13.7–43.0	23.0	24.7	25.6	30.9	34.2	38.8	40.2
≥85	<161	29 (4.6)	19.1 (4.6)	16.2	6.2–30.6	13.5	14.5	17.4	19.1	21.5	22.6	25.2
	[161–167[	16 (2.5)	23.9 (6.2)	20.3	12.9–36.5	14.7	16.3	19.8	24.5	27.4	30.3	34.5
	≥167	11 (1.8)	29.2 (9.0)	24.8	21.2–46.0	21.2	21.3	21.3	26.1	32.8	45.8	45.9

*Abbreviations: n* number of subjects, *P* percentile, *SD* standard deviation

**Fig. 1:**
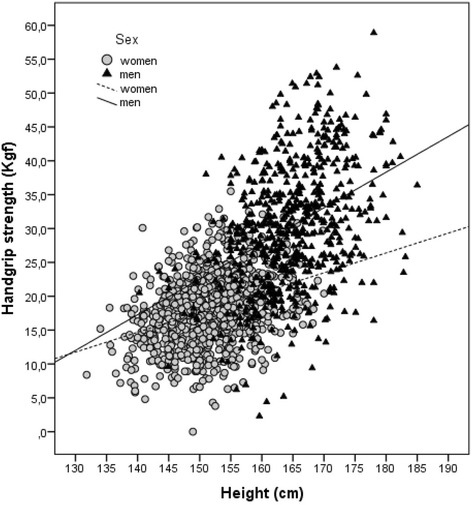
Correlation between handgrip strength and height, in women (R^2^ linear = 0.11) and in men (R^2^ linear = 0.15)

Age (*r* = − 0.44, in women; *r* = − 0.42, in men), cognitive status (*r* = 0.32, in women; *r* = 0.37, in men), self-reported sitting time (*r* = − 0.38, in women; *r* = − 0.34, in men), practice of physical activity (*r* = 0.37, in women; *r* = 0.31, in men) and MNA-SF (*r* = 0.19, in women; *r* = 0.16, in men) were significantly correlated (*p* < 0.001) with HGS values (Table 3). Height was the anthropometric parameter most correlated with HGS (*r* = 0.34, *p* < 0.001, in women and *r* = 0.40, *p* < 0.001, in men) (Table 3 and Fig. 1).

**Table 3:** Correlations between handgrip strength and other variables

	Women		Men	
Variables	Pearson correlation coefficient	*p*	Pearson correlation coefficient	*p*
Age (years)	- 0.44	<0.001	- 0.42	<0.001
Mini-Mental State Examination (score)	0.32	<0.001	0.37	<0.001
Number of chronic diseases (n)	- 0.12	0.002	- 0.03	0.561
Self-reported sitting time (hours/day)	- 0.38	<0.001	- 0.34	<0.001
Practice of physical activity (hours/day)	0.37	<0.001	0.31	<0.001
Weight (Kg)	0.02	0.621	0.01	0.879
Height (cm)	0.34	<0.001	0.40	<0.001
Body mass index (Kg/m^2^),	- 0.04	0.194	- 0.04	0.385
Mid-arm muscle circumference (cm)	0.05	0.125	0.19	<0.001
Waist circumference (cm)	- 0.06	0.093	- 0.03	0.422
Calf circumference (cm)	0.19	<0.001	0.02	0.675
Mini-Nutritional Assessment (score)	0.19	<0.001	0.16	<0.001

**Fig. 2:**
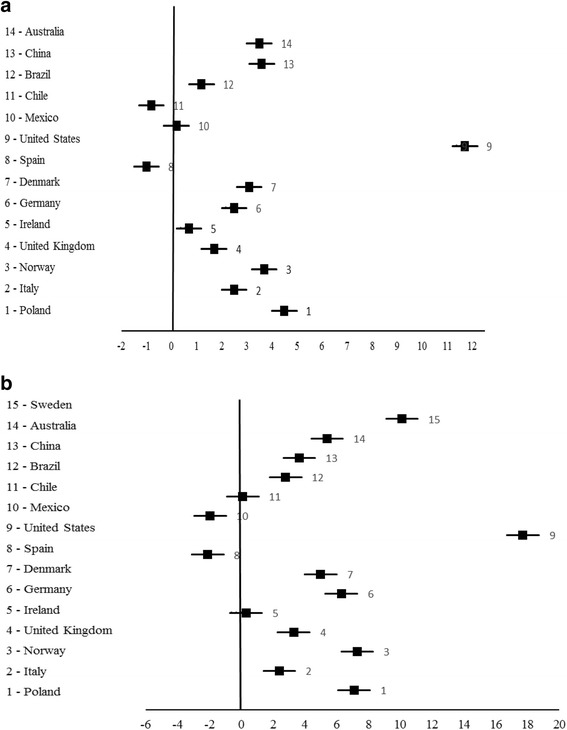
Handgrip strength mean differences in Kgf (95% CI), between other older populations and Portuguese older adults (**a** – women; **b** – men)

**Fig. 3:**
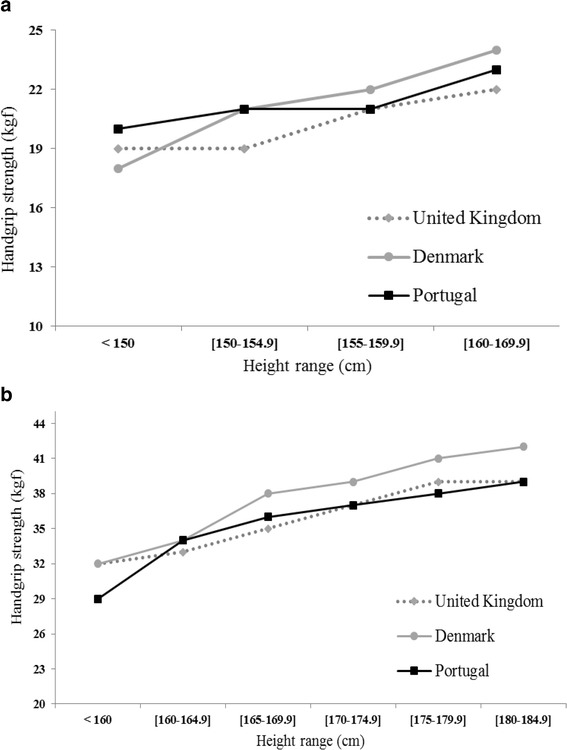
Mean or median values of handgrip strength, according to height range, in women (**a**) and in men (**b**), aged 65 to 75 years - comparison between different populations

Information about HGS mean values of older adults from different countries are presented in Table 4. In general, HGS values of Portuguese older adults were lower than other older populations. However, present sample presented higher HGS mean values when compared to Spanish and Chilean women, as well as Spanish and Mexican men (Fig. 2). Despite United Kingdom older adults presenting higher HGS values than the Portuguese sample, they were closer when the results are presented stratified by height (Fig. 3).

**Table 4:** Measurement characteristics and results of handgrip strength in community-dwelling older adults, according to studies conducted in different populations

Study (year)	Country	Age range (years)	Type of dynamometer	Measurement/hand examined	Mean value of handgrip strength (Kgf)	
					Women	Men
Leong et al. [20] (2016)	Sweden and Poland (2456)	[61–70]	Jamar hydraulic	Maximum value of three measurements/both hands	25.0^(b)^	41.0^(b)^
Veronese et al. [21] (2016)	Italy (2096)	[65–103]	Jamar hydraulic	Maximum value of three measurements/both hands	20.5^(b)^	32.7^(b)^
Tveter et al. [22] (2014)	Norway (159)	[61–70]	Hydraulic dynamometer (not specify)	Mean of two measurements/ both hands	24.3 (L) 24.1 (R)	41.2 (L) 41.2 (R)
Spruit et al. [23] (2013)	United Kingdom^(a)^	[65–73]	Jamar hydraulic	Mean of three measurements/ both hands	20.9 (L) 23.1 (R)	35.9 (L) 38.0 (R)
Kenny et al. [24] (2013)	Ireland^(a)^	[65–85]	Baseline hydraulic	Maximum value of two measurements/both hands	19.2 ^(b)^	31.3^(b)^
Ribom et al. [25] (2011)	Sweden (999 men)	[70–80]	Jamar hydraulic	Mean of two measurements/ both hands	----	40.2 (L) 41.1 (R)
Günther et al. [26] (2008)	Germany (258)	[60–95]	Baseline hydraulic	Mean of three measurements/ both hands	20.0 (L) 21.0 (R)	35.3 (L) 38.0 (R)
Frederiksen et al. [27] (2006)	Denmark^(a)^	[65–75]	Smedley mechanical	Maximum value of three measurements/both hands	23.0^(b)^	38.0^(b)^
Luna-Heredia et al. [28] (2005)	Spain (225)	[65–97]	Baseline and Grip-D dynamometer	Maximum value of three measurements/both hands	16.0 (ND) 17.9 (D)	27.1 (ND) 29.4 (D)
				Mean of three measurements/ both hands	15.1 (ND) 16.7 (D)	25.6 (ND) 27.8 (D)
Cote et al. [29] (2014)	United States (95)	≥ 60	Jamar hydraulic	Maximum value of measurements/both hands	29.2 (L) 30.1 (R)	46.1 (L) 50.0 (R)
Al Snih et al. [30] (2002)	Mexico (2488)	≥ 65	Jamar hydraulic	Maximum value of two measurements/dominant hand	18.2 (D)	28.4 (D)
Mancilla et al. [31] (2016)	Chile (1047)	[60–91]	Jamar hydraulic	Maximum value of two measurements/both hands	16.8 (L) 17.7 (R)	29.9 (L) 31.2 (R)
Schlüssel et al. [32] (2008)	Brazil (567)	≥ 60	Jamar hydraulic	Maximum value of three measurements/both hands	18.7 (L) 19.7 (R)	32.0 (L) 34.3 (R)
Zeng et al. [33] (2016)	China (461)	≥ 60	Hand dynamometer (WCS-II, Beijing)	Maximum value of two measurements/both hands	21.6 ^(b)^	34.0 ^(b)^
Massy-Westropp et al. [34] (2011)	Australia ^(a)^	≥ 60	Jamar hydraulic	Mean of three measurements/both hands	21.0 (L) 22.0 (R)	35.0 (L) 36.5 (R)

*Abbreviations*: *D* dominant hand, *L* left hand, *ND*non-dominant hand, *R* right hand

^a^Number of subjects was not available for the age group considered

^b^The study only reported the mean value of both hands

## Discussion

Several HGS values, resulting from populations with different nationalities and ethnicities, have been published in the literature cited as reference values [20–34], however for the Portuguese population HGS values had never been described. In addition, HGS of older adult populations, despite their particular characteristics, are not always analyzed separately from adult populations. The grouping of strength values together for all older adults was also referred as a limitation in Bohannon et al. meta-analysis, failing to take into consideration the continued decline in muscle strength that occurs as individuals age [35]. This problem was overcome by the report of data for strata of age after 75 years, but restricted to samples from confined geographic regions of United States [36].

Overall, Portuguese older adults had lower HGS values than other older populations. A wide range of factors, including individual characteristics and those related to different nationalities and ethnicities may have influenced these results.

The present study included individuals classified with cognitive impairment according to the Portuguese version of the MMSE and a decreased grip strength is strongly associated with developing mild cognitive impairment [37]. Mild cognitive impairment is a transitional state that goes beyond typical age-related cognitive changes, but is marked by less severe impairment than dementia [38]. The association between HGS and mild cognitive impairment could be due to several mechanisms. Both motor and cognitive performance rely upon the nervous system to execute activity, thus, a compromised nervous system (e.g., as a result of inflammation) may lead to general deficits in both areas [39]. On the other hand, some degree of cognitive impairment is common in the ageing process and the non-exclusion of those participants decreased the risk of a selection bias [1, 40]. In addition, although 9.5% of the participating older adults presented cognitive impairment, they were included only if they were able to understand the instructions and to undertake the HGS technique correctly, in order to avoid inclusion of cases with true underlying neurodegenerative processes. Nevertheless, additional clinical diagnostic tools would be necessary to confirm the stage of the cognitive impairment and also to infer about these results.

Another point is that our sample presented a very high proportion of participants who reported chronic diseases, in particular arthrosis, cervical and lumbar pain. However, according to Onder et al., daily pain is highly prevalent among community-dwelling older adults, despite being associated with impaired muscle strength [41]. A long sitting time period, as well few hours of practice of physical activity per day were also factors that can justify low HGS values in our study, since these parameters are potential independent health indicators among older adults [42].

Nutritional impairment has been closely associated with low HGS values [43] and in this sample, 14.8% of participants were at nutritional risk and 1.3% undernourished. However, this proportion of older adults at nutritional risk was similar to those previously reported, which does not explain the low HGS values [44].

Simultaneously, overweight identified through the World Health Organization criteria, was highly frequent among participants of both sexes [45]. Although overweight and obesity have also been associated with reduced strength [46], this association was not significant in the present work. Body mass index reflect the ratio of weight to height, and there was also no significant association between weight and HGS. It is important to emphasize that total weight use as a surrogate indicator of adiposity can be prone to errors, because fat mass and fat free mass are not distinguished [47]. In addition, according to some epidemiological evidence, older populations display higher optimal BMI intervals than younger people [47]. These facts can induce confounding in understanding association between BMI and HGS.

On the other hand, height was directly and significantly associated with HGS in women and men. Accordingly, in Han et al. study, skeletal muscle mass adjusted by height correlated better with muscular functions and HGS, than that adjusted for body weight [48]. Height reflects part of the bone structure, and bone mass has also demonstrated implications for muscular and strength performances [26, 48]. The International Working Group on Sarcopenia recommends the inclusion of height value for determining relative muscle mass, in a context of functional disability [49]. The effect of skeletal muscle mass on HGS values should not be interpreted alone, but together with other structural and anatomic measurements, including the hand anatomy [50].

In addition, although in overall HGS of United Kingdom sample were higher than HGS of Portuguese sample, Portuguese HGS values were more similar to United Kingdom values when stratified by height. This fact reinforces the importance of height adjustment in the interpretation of HGS results.

Indeed, different HGS mean values were observed according to different countries, but this fact has also been reported by other studies [20] and the nature of these differences has not yet been resolved [20]. However, heterogeneous designs may explain some of the differences among studies results. For example, in data from Danish older adults, HGS decline with ageing was presented in the end of the follow-up [27]. On the contrary, in the other analyzed studies, including the present report, HGS data was obtained from cross-sectional designs [22, 23, 26, 28, 29, 31–33] or from cross-sectional analysis of cohorts [20, 21, 24, 25, 30, 34].

As expected, HGS values of populations from Chile, Mexico and Spain were closer to present data than HGS values of those from United States, China or Australia. Nevertheless, Chilean and Spanish studies were based on convenience samples [28, 31] and the lack of representation can justify some deviation in the mean of HGS comparing to our study. In addition, in Chilean, Spanish and Brazilian studies [28, 31, 32], as well as in studies from United States and China [29, 33], potential comorbidities of participants were not described. Otherwise, in studies from Sweden, United Kingdom, Poland and Australia [20, 23, 25, 34], participants with chronic diseases, such as osteoarthritis and inflammatory disease, were excluded and this fact can explain, in part, their higher HGS values in comparison to present study.

Handgrip strength of Italian population was also slightly higher than HGS of Portuguese sample, but is important to note that Veronese et al. presented HGS results for the mean value of both hands [21] while results for the non-dominant hand presented here. Non-dominant hand HGS measurement were used because values of the dominant hand are more affected by occupational load than values of the non-dominant [19]. Consequences of occupational load can induce localized muscular development or an excessive articular contact stress and so, the non-dominant hand can express more independently the overall muscle strength [19].

It is also uncertain which of the two factors, genetic or environment, are more decisive to the HGS results [20]. Besides ethnic differences in height and in skeletal muscle mass and function [9], there are well-recognized differences in dietary protein intake according to different countries, and the variation in dietary patterns may also explain differences in muscle strength [51]. Education and socio-economic status are other factors that can explain differences in HGS ranges among countries [20, 52, 53].

In present analysis, individuals from the United States showed the highest HGS values and Spanish, followed by Chilean and Mexican individuals, showed the lowest HGS values. This is in agreement with results from a recent study in which grip strength data from regions in South America were clearly low compared to developed regions of the United States [54]. In a systematic review about associations between HGS and sociodemographic factors, older adults with lower HGS values had lower educational levels [52]. Accordingly, in our sample 14% of participants had never attended school, as well as 77% of them had only between one and four completed school years. Countries with contrasting income may therefore be expected to be associated with wider discrepancies in HGS values [20, 52, 53].

The main strength of this work was to describe, for the first time, HGS values of the Portuguese population aged ≥65 years in a nationwide representative sample. Handgrip strength reference values of at least 85% of the mean of the population are used to describe normality [28]. However, the values of our sample were frequently below the cut-off of 85% of the HGS mean due to changes related to the aging process and were similar to those associated with weakness and mobility limitation (<16 Kgf in women and <26 Kgf in men), according to the Foundation for the National Institutes of Health Sarcopenic Project [49]. This Portuguese older population presented very low HGS values. Considering this fact together the high frequency of overweight, obesity and chronic disabilities, these first results are a matter of concern indicating the need of public health interventions. In addition, as a wide range of variables may influence HGS, reflection is required on how normative HGS values should be constructed for this age group and which variables should be considered in their definition.

The comparison between HGS data published in the literature is hampered by the heterogeneous study designs and by heterogeneous characteristics of the samples and of the methodology used. Regarding to HGS measurements, some authors reported only “left” or “right” hand, without referring to the hand dominance [22, 23, 25, 26, 29, 31, 32, 34], and in other cases only the average of both hands was referred [20, 21, 24, 27, 33], introducing some bias in data analysis. The importance of a consensus on which hand HGS measurements should be performed, is herein reinforced, to improve data comparison in future works. In addition, in some studies, the lowest age range included subjects aged 60 years and over, while in others, the lowest age was 65 years. The maximum age of participants was also not reported in five of the 15 analyzed studies [29, 30, 32–34]. Therefore, a bias related to the differences in age groups may has been introduced in the comparison of HGS values.

## Conclusions

This study described, for the first time, HGS values of Portuguese population aged ≥65 years, in accordance with age and with sex-specific tertiles of height. The definition of HGS reference values in this age group merits further reflection.

## Abbreviations

BMI: Body mass index
HGS: Handgrip strength
MMSE: Mini Mental State Examination
MNA-SF: Mini-Nutritional Assessment - Short Form
